# Annexin A4 Is Dispensable for Hair Cell Development and Function

**DOI:** 10.3389/fcell.2021.680155

**Published:** 2021-06-03

**Authors:** Nana Li, Yuehui Xi, Haibo Du, Hao Zhou, Zhigang Xu

**Affiliations:** ^1^Shandong Provincial Key Laboratory of Animal Cell and Developmental Biology, School of Life Sciences, Shandong University, Qingdao, China; ^2^Shandong Provincial Collaborative Innovation Center of Cell Biology, Shandong Normal University, Jinan, China

**Keywords:** *Anxa4*, inner ear, hair cells, stereocilia, knockout mice

## Abstract

Annexin A4 (ANXA4) is a Ca^2+^-dependent phospholipid-binding protein that is specifically expressed in the cochlear and vestibular hair cells, but its function in the hair cells remains unknown. In the present study, we show that besides localizing on the plasma membrane, ANXA4 immunoreactivity is also localized at the tips of stereocilia in the hair cells. In order to investigate the role of ANXA4 in the hair cells, we established *Anxa4* knockout mice using CRISPR/Cas9 technique. Unexpectedly, the development of both cochlear and vestibular hair cells is normal in *Anxa4* knockout mice. Moreover, stereocilia morphology of *Anxa4* knockout mice is normal, so is the mechano-electrical transduction (MET) function. Consistently, the auditory and vestibular functions are normal in the knockout mice. In conclusion, we show here that ANXA4 is dispensable for the development and function of hair cells, which might result from functional redundancy between ANXA4 and other annexin(s) in the hair cells.

## Introduction

Hair cells are mechanosensitive sensory receptor cells in the cochlea and vestibular organs, characterized by their hairy-looking, actin-based stereocilia on the apical surface. The stereocilia are organized into several rows of increasing height, forming a staircase-like pattern. Mechanical stimuli causes deflection of the stereocilia, which changes the opening probability of the mechano-electrical transduction (MET) channels localized at the tips of shorter row stereocilia, resulting in influx of cations into the hair cells (Hudspeth and Jacobs, 1979; Beurg et al., 2009; Schwander et al., 2010). The development and function of hair cells are tightly regulated, and deficits in this process are the main reasons for hearing loss and balancing dysfunction (Müller and Barr-Gillespie, 2015).

Genetic, transcriptomic and proteomic analysis have identified several proteins that are essential for hair cell development and/or function (Richardson et al., 2011; Cai et al., 2015; Scheffer et al., 2015; Krey et al., 2018). Some of the identified proteins are specifically expressed in the hair cells, such as ATOH1, a famous transcription factor that plays pivotal roles in hair cells. *Atoh1*-deficient mice fail to generate hair cells, and overexpression of *Atoh1* in immature rodent inner ears induce ectopic hair cells, suggesting that ATOH1 is important for hair cell formation during development (Bermingham et al., 1999; Zheng and Gao, 2000; Woods et al., 2004). Several ATOH1-regulated genes have been identified through transcriptomic analysis, such as *Anxa4*, *Rbm24*, *Srrm4*, et al. (Cai et al., 2015). Previously, we and others showed that RBM24 and SRRM4 are important for hair cell development and/or function through regulating mRNA splicing or stability (Nakano et al., 2012; Zhang et al., 2020; Zheng et al., 2020).

*Anxa4* encodes annexin A4 (ANXA4), a Ca^2+^-dependent phospholipid-binding protein that is predominantly expressed in the epithelial cells (Kaetzel et al., 1989, 1994). Upon Ca^2+^ binding, ANXA4 undergoes oligomerization and translocates to the plasma membrane (Crosby et al., 2013). ANXA4 binding increases the rigidity of plasma membrane, induces membrane curvature, and reduces the permeability of water, H^+^, and Cl^–^ (Kaetzel et al., 1994, 2001; Hill et al., 2003; Boye et al., 2017). Moreover, ANXA4 was suggested to be a direct regulator of adenylyl cyclase type 5 (AC5) (Heinick et al., 2015). In the mouse cochlea, *in situ* hybridization reveals that *Anxa4* is expressed in the outer hair cells (OHCs) and inner hair cells (IHCs) (Cai et al., 2015). In the mouse vestibular organs, immunostaining suggests that ANXA4 is expressed at the plasma membrane of type II vestibular hair cells (VHCs) (McInturff et al., 2018). The hair cell-specific expression of *Anxa4* is further supported by the transcriptome data (Scheffer et al., 2015; Shen et al., 2015). However, despite of the specific expression pattern, little is known about the function of ANXA4 in the hair cells.

Three *Anxa4* transcripts have been identified in mice, which have different 5′ untranslated regions (5′UTR) but encode an identical ANXA4 protein (Li et al., 2003). An *Anxa4* gene trap mouse line was created through inserting a retrovirus-based trapping cassette into the first intron of *Anxa4* gene, which disrupts the expression of one *Anxa4* transcript but leaves the other two unaffected (Li et al., 2003). In the present work, we establish *Anxa4* knockout mice by deleting exons 3–6 of *Anxa4* gene that are common to all three *Anxa4* transcripts. Using this knockout mouse model, we investigate the role of ANXA4 in the development and function of hair cells.

## Materials and Methods

### Mice

*Anxa4* knockout mice were generated using clustered regularly interspaced short palindromic repeats (CRISPR)/Cas9 technique by Cyagen Biosciences Inc. (Suzhou, China) on a C57BL/6N background. Briefly, genomic DNA sequences 5′-TGTTATAAATATAACGCACAAGG-3′ and 5′-AGCCTGAGCCTACACCTCGAGGG-3′ were chosen as the guide RNA (gRNA) targets. *In vitro* transcribed gRNAs and *Cas9* mRNA were injected into the cytoplasm of zygotes, followed by culturing for 24 h *in vitro*. The injected zygotes were transferred into the oviduct of a pseudopregnant ICR female mouse at 0.5 day post coitus (dpc) to give rise to F0 mice, which were then crossed to wild type C57BL/6N mice to give rise to heterozygous F1 mice. The following genotyping primers were used for examination of the knockout allele: F1, 5′-AGATCCCCATCCAAATAAGGTTTG-3′; R1, 5′-ATCACTTTACAGAGTTTCGA-3′ (436 bp). The following genotyping primers were used for examination of the wild type allele: F2, 5′-CTTAGGACTGACTTCCTGTGTCAT-3′; R2, 5′-TTGTCCATACAGCAATGAAGGGAT-3′ (620 bp). *Atoh1-GFP* transgenic mice and *Cdh23-V^2J^* mice were maintained and genotyped as described previously (Di Palma et al., 2001; Lumpkin et al., 2003).

### Reverse Transcription-Polymerase Chain Reaction (RT-PCR)

Total RNA of mouse inner ear was extracted using TRIzol reagent (Invitrogen) according to the manufacturer’s protocol. Afterward, 1 μg total RNA was used for reverse transcription (RT) using PrimeScript RT Reagent Kit with gDNA Eraser (Takara, RR047A). Polymerase chain reaction (PCR) was then performed using the RT product as template with the following primers: *Anxa1*-F, 5′-ATGTATCCTCGGATGTTGCTGC-3′; *Anxa1*-R, 5′-TGAGCATTGGTCCTCTTGGTA-3′; *Anxa2*-F, 5′-ATGTCTACTGTCCACGAAATCCT-3′; *Anxa2*-R, 5′-CGAAG TTGGTGTAGGGTTTGACT-3′; *Anxa4*-F, 5′-TGTGACTGAA CTCTGAACGTGA-3′; *Anxa4*-R, 5′-TTTCACCTCGTCTGTC CCCC-3′; *Anxa5*-F, 5′-ATCCTGAACCTGTTGACATCCC-3′; *Anxa5*-R, 5′-AGTCGTGAGGGCTTCATCATA-3′; *Anxa6*-F, 5′-CCTATTGTGACGCCAAAGAGAT-3′; *Anxa6*-R, 5′-GCCGG AAGTGTCTCCAATGA-3′; *Anxa7*-F, 5′-AGGATTGTGGTCA CTCGAAGT-3′; *Anxa7*-R, 5′-TGTAATCTCCACTCGTG TCACT-3′; β*-actin*-F, 5,-CGTTGACATCCGTAAAGACC-3′; β*-actin*-R, 5′-AACAGTCCGCCTAGAAGCAC-3′. Annealing temperatures were adjusted between 58 and 62°C to obtain the optimal sensitivity and specificity.

### Western Blot

Mouse tissues were dissected and lysed in ice-cold lysis buffer containing 150 mM NaCl, 50 mM Tris at pH 7.5, 1% (vol/vol) Triton X-100, 1 mM PMSF, and 1 × protease inhibitor cocktail (Sigma-Aldrich, Cat. No. S8830). After centrifugation, the supernatant was separated by polyacrylamide gel electrophoresis (PAGE), followed by transferring to PVDF membrane. The membrane was blocked in PBS with 5% BSA and 0.1% Tween-20, followed by incubation with primary antibody at 4°C overnight and corresponding secondary antibody at room temperature for an hour. The signals were detected using the ECL system (Thermo Fisher Scientific). Antibodies used in the present study are as follows: rabbit anti-ANXA4 antibody (ABclonal, Cat. No. A13466); mouse anti-GAPDH antibody (Millipore, Cat. No. MAB374); HRP-conjugated goat anti-mouse antibody (Bio-Rad, Cat. No. 170-6516); HRP-conjugated goat anti-rabbit antibody (Bio-Rad, Cat. No. 170-6515).

### Whole-Mount Immunostaining

Whole-mount immunostaining was performed as previously described (Du et al., 2020). Briefly, dissected sensory epithelia were fixed with 4% paraformaldehyde (PFA) in PBS for 30 min, then permeabilized and blocked with PBT1 (0.1% Triton X-100, 1% BSA, and 5% heat-inactivated goat serum in PBS, pH 7.3) for an hour. The samples were then incubated with primary antibody in PBT1 overnight at 4°C, followed by incubation with secondary antibody in PBT2 (0.1% Triton X-100 and 0.1% BSA in PBS) for an hour. For stereocilia visualization, samples were additionally treated with TRITC-conjugated phalloidin (Sigma-Aldrich, Cat. No. P1951) in PBS for 30 min. The samples were mounted in PBS/glycerol (1:1) and imaged with confocal microscope (LSM 700 and 900, Zeiss, Germany). Antibodies used in the present study are as follows: goat anti-ANXA4 antibody (R&D Systems, Cat. No. AF4146); rabbit anti-ANXA4 antibody (ABclonal, Cat. No. A13466); rabbit anti-MYO7A antibody (Proteus Biosciences, Cat. No. 25-6790); Alexa Fluor 488-conjugated donkey anti-rabbit IgG (Thermo Fisher Scientific, Cat. No. A21206); Alexa Fluor 488-conjugated donkey anti-goat IgG (Thermo Fisher Scientific, Cat. No. A11055); Cy3 rabbit anti-goat IgG (H + L) (ABclonal, Cat. No. AS015).

### Scanning Electron Microscopy (SEM)

The temporal bone were fixed with 2.5% glutaraldehyde in 0.1 M phosphate buffer overnight at 4°C, then the cochlea were dissected out and post-fixed with 1% osmium tetroxide in 0.1 M phosphate buffer at 4°C for 2 h. After that, samples were dehydrated in ethanol and critically point dried using a Leica EM CPD300 (Leica, Germany). Samples were then mounted and sputter coated with platinum (15 nm) using a Cressington 108 sputter coater (Cressington, United Kingdom), and images were taken using a Quanta250 field-emission scanning electron microscope (FEI, Netherlands) with a beam strength of 3 kV.

### Auditory Brainstem Response (ABR) Measurement

RZ6 workstation and BioSig software (Tucker-Davis Technologies, Alachua, FL, United States) were used to measure and analyze ABR. Mice were anesthetized by intraperitoneally injecting Pentobarbital (8.4 mg/100 g body weight) and placed on an isothermal pad to keep the body temperature during the experiment. Acoustic stimuli (clicks or pure-tone bursts) of decreasing sound level from 90 dB SPL in 10 dB SPL steps were delivered to the mouse ear through a loudspeaker (MF1, Tucker-Davis Technologies). A total of 512 responses were sampled and averaged at each sound level. Hearing threshold was determined as the lowest sound level at which the ABR waves were recognizable.

### Vestibular Function Examination

Vestibular function of mice was evaluated as described previously (Fasquelle et al., 2011). Briefly, six different tests were performed as follows: (1) Head bobbing was counted to record abnormal intermittent backward extension of the neck; (2) Retropulsion was counted to record typical backward walk due to vestibular disturbance; (3) Circling stereotyped movement was counted to record compulsive circles around the animal’s hips; (4) Swimming test was performed to observe swimming behavior ranging from normal swimming to drowning; (5) Tail-hanging reflex was tested to examine the normal forelimb extension to reach the ground; (6) Rotarod test was performed to evaluate the falling latency on rotating rod. For tests of head bobbing, retropulsion and circling stereotyped movement, mice were placed in a 45 × 30 cm cube for 1 min and the frequency of corresponding behaviors was recorded. Swimming test scores were defined as follows: 0, normal swimming; 1, irregular swimming; 2, immobile floating; and 3, underwater tumbling. For tail hanging reflex test, mice were scored 0–4 corresponding to normal to severe vestibular defect. For rotarod test, the rod apparatus (HB-600, Ruanlong, China) was set to accelerate from 0 to 50 rpm over a 3-min period. Mice were placed on the rod and the time before dropping from the rod was measured automatically. Mice were trained for four consecutive days and testing data was recorded on day 2, 3, and 4. Each day consisted of four trials and the second, third and fourth one was measured with the first one as a training trial.

### FM1-43FX Uptake Experiment

Mouse auditory or vestibular sensory epithelia were dissected out and incubated with 3 μM FM 1-43FX (Thermo Fisher, Cat. No. F35355) in PBS for 30 s, followed by washing three times with PBS. The samples were then fixed with 4% PFA at room temperature for 20 min, and mounted in PBS-glycerol (1:1). Images were taken using a confocal microscope (LSM 700, Zeiss, Germany).

### Statistical Analysis

All experiments were performed at least three times independently. Data were shown as means ± standard error of mean (SEM). Student’s two-tailed unpaired *t*-test was used to determine statistical significance, and *P* < 0.05 was considered statistically significant.

## Results

### ANXA4 Is Localized at the Stereociliary Tips and Plasma Membrane of Cochlear Hair Cells

**FIGURE 1:**
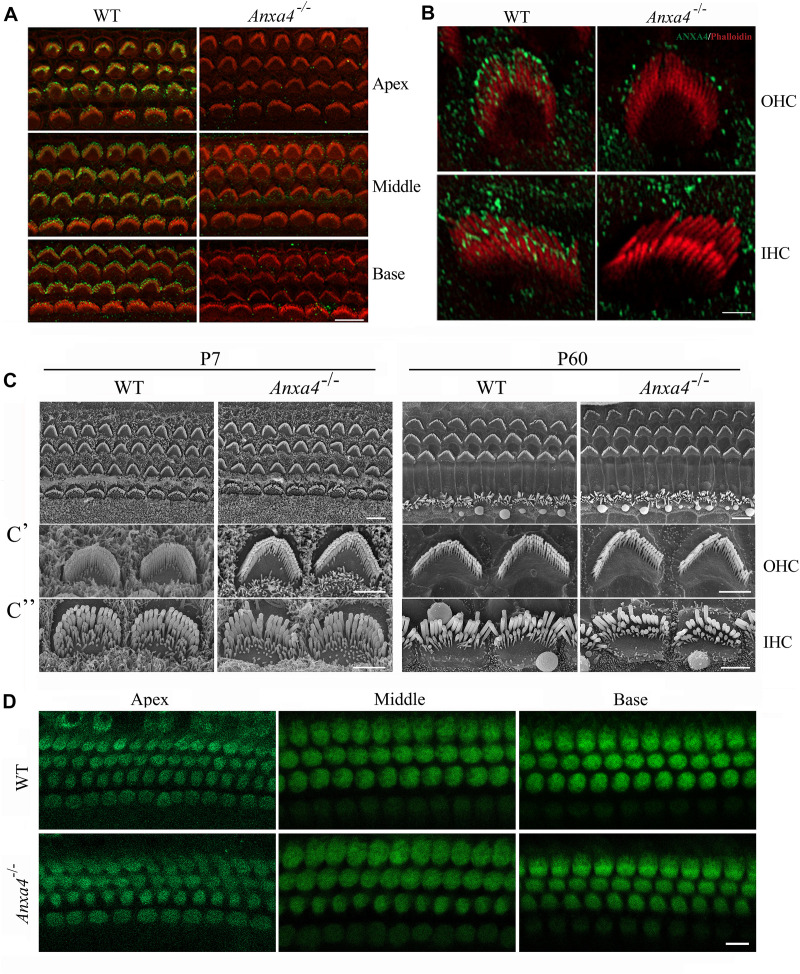
Stereocilia morphology and MET function of cochlear hair cells are unaffected in *Anxa4^–/–^* mice. **(A)** Cochlear hair cell stereocilia of P5 wild type or *Anxa4^–/–^* mice were stained with anti-ANXA4 antibody (ABclonal) and imaged using confocal microscope. Stereocilia F-actin core was visualized by staining with TRITC-conjugated phalloidin. **(B)** Higher resolution confocal microscopy images show stereociliary tip localization of ANXA4 in P7 OHC and IHC. **(C)** Examination of hair bundle morphology of wild type or *Anxa4^–/–^* mice at P7 or P60 using SEM. **(C’,C”)** Higher resolution SEM images of OHCs and IHCs, respectively. **(D)** FM1-43FX uptake by OHCs and IHCs of P7 wild type or *Anxa4^–/–^* mice was examined using confocal microscope. Shown are images taken from the middle turn unless otherwise indicated. Scale bars, 10 μm in **(A,D)**, 2 μm in **(B**,**C’,C”)**, 5 μm in **(C)**.

**FIGURE 2:**
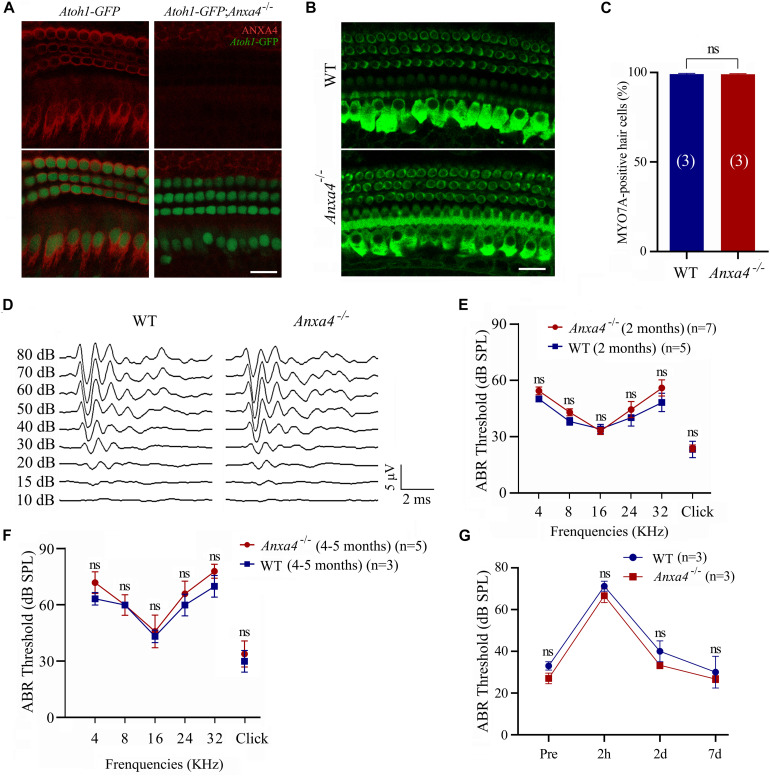
Cochlear hair cells and auditory function are unaffected in *Anxa4^–/–^* mice. **(A)** Cochlear hair cells of P7 *Atoh1-GFP* or *Atoh1-GFP*; *Anxa4^–/–^* mice were stained with anti-ANXA4 antibody (R&D Systems) and imaged using confocal microscope. Hair cells were labeled by nuclear GFP. **(B)** Cochlear hair cells of P60 wild type or *Anxa4^–/–^* mice were stained with anti-MYO7A antibody and imaged using confocal microscope. Shown are images taken from the middle turn. **(C)** Numbers of MYO7A-positive hair cells from different genotypes as indicated were calculated according to the results from **(B)**. **(D)** Raw traces of ABR responses to click stimuli in 2-month-old wild type or *Anxa4^–/–^* mice. **(E)** The ABR thresholds for pure tone and click stimuli in 2-month-old wild type or *Anxa4^–/–^* mice. **(F)** The ABR thresholds for pure tone and click stimuli in 4–5-month-old wild type or *Anxa4^–/–^* mice. **(G)** Two-month-old wild type or *Anxa4^–/–^* mice were subjected to 2–20 kHz noise at 110 dB DSL for 2 hours, and ABR thresholds were measured pre-exposure and at different post-exposure time points as indicated. The numbers of animals used in each experiments are indicated in brackets. Scale bars, 20 μm in **(A,B)**. ns, not significant.

We first examined the localization of ANXA4 in the cochlear hair cells by performing whole-mount immunostaining using a commercial anti-ANXA4 antibody. The results reveal robust ANXA4 immunoreacivity in the stereocilia of both OHCs and IHCs (Figure 1A). Further examination at higher resolution shows that ANXA4 immunoreacivity is enriched at the stereociliary tips of OHCs and IHCs (Figure 1B). Similar results were obtained using another commercial anti-ANXA4 antibody (Supplementary Figures 1A–C). The specificity of the antibodies were confirmed using *Anxa4* knockout mice (see below) (Figures 1A,B and Supplementary Figures 1A–C). Moreover, ANXA4 immunoreactivity is also detected on the plasma membrane of OHCs and IHCs (Figure 2A). Taken together, our present data suggest that ANXA4 is localized at the stereociliary tips and plasma membrane of cochlear hair cells.

### Generation of *Anxa4* Knockout Mice

Mouse *Anxa4* gene contains 13 exons, with the start codon ATG localized in exon 2 and stop codon TAA localized in exon 13 (Supplementary Figure 2A). Transcription could start from exon 1 or exon 2, or an alternative exon 1 (exon 1’), giving rise to three different transcripts encoding an identical ANXA4 protein (Li et al., 2003) (Supplementary Figure 2A). Two gRNAs were used to delete exons 3–6, which are common for all three *Anxa4* transcripts (Supplementary Figure 2A). This deletion is expected to cause a premature translational stop and give rise to a potentially truncated ANXA4 protein (Supplementary Figure 2B).

RT-PCR was performed to examine the expression of *Anxa4* transcript using primers flanking the deleted region, which gives rise to two PCR products in *Anxa4^–/–^* mice (Supplementary Figure 2C). Sanger sequencing reveals that the smaller band represents the expected *Anxa4* transcript with exon 3–6 deleted, whereas the larger band represents a similar transcript with additional 43 nucleotide acids corresponding to sequences from intron 2 (Supplementary Figure 2D). The smaller knockout transcript will generate a peptide of 41 amino acids (aa) unrelated to ANXA4, while the larger knockout transcript might produce a truncated ANXA4 protein containing the last two ANX domains (Supplementary Figure 2B). Nevertheless, the expression level of these two knockout transcripts is much lower than that of the wild type *Anxa4* transcript, suggesting that they undergo non-specific mRNA decay (NMD) (Supplementary Figure 2C).

ANXA4 has been reported to have a molecular weight of 35 kDa (Kaetzel et al., 1994; Li et al., 2003). Consistently, western blot detects a band of 35 kDa from *Anxa4*^+/–^ mice but not *Anxa4^–/–^* mice, suggesting that ANXA4 expression is completely disrupted in the *Anxa4* knockout mice (Supplementary Figure 2E). *Anxa4^–/–^* mice are morphologically and behaviorally indistinguishable from *Anxa4*^+/–^ or wild type mice. Moreover, interbreeding of *Anxa4*^+/–^ mice gives rise to offspring in the expected Mendelian ratio (21% wild-type, 49% *Anxa4*^+/–^, and 30% *Anxa4^–/–^*; n = 86; χ^2^-test *P*-value = 0.68) with normal viability. These results suggest that ANXA4 is dispensable for general development in mice.

### Stereocilia Development and MET Function Are Unaffected in *Anxa4* Knockout Mice

Phalloidin staining shows that stereocilia morphology is normal in *Anxa4^–/–^* mice (Figures 1A,B and Supplementary Figures 1A–C). Stereocilia morphology was further examined by performing scanning electron microscopy (SEM), which shows that the stereocilia in both OHCs and IHCs of *Anxa4^–/–^* mice are indistinguishable from that of wild type mice up to postnatal day 60 (P60) (Figures 1C–C”). Therefore, our present data suggest that *Anxa4* disruption does not affect the development or maintenance of stereocilia.

It has long been known that stereocilia play a pivotal role in MET of hair cells (Hudspeth and Jacobs, 1979). The normal morphology of stereocilia suggest that MET function might also be unaffected in *Anxa4^–/–^* mice. MET function was then examined by performing FM1-43FX uptake experiment. FM1-43FX is a fluorescent dye that enters hair cells through the MET channels when applied briefly, hence is used as an indicator of MET function of hair cells (Gale et al., 2001; Meyers et al., 2003). Consistent with the normal stereocilia morphology, FM1-43FX uptake is normal in neonatal *Anxa4^–/–^* cochlear hair cells, suggesting that MET is unaffected by *Anxa4* disruption (Figure 1D).

### Cochlear Hair Cells and Auditory Function Are Unaffected in *Anxa4* Knockout Mice

To facilitate examination of the inner ear hair cells, we crossed *Anxa4* knockout mice with *Atoh1-GFP* transgenic mice that express a nuclear GFP driven by *Atoh1* enhancer (Lumpkin et al., 2003). As previously reported, GFP strongly labels the nuclei of cochlear hair cells in this transgenic line (Figure 2A). Immunostaining shows robust ANXA4 immunoreactivity on the plasma membrane of IHCs and OHCs in *Atoh1-GFP* mice but not *Atoh1-GFP*; *Anxa4^–/–^* mice at P7 (Figure 2A). However, no loss of GFP-positive cochlear hair cells is observed in *Atoh1-GFP*; *Anxa4^–/–^* mice at this age (Figure 2A). Cochlear hair cells were further examined in 2-month-old mice by immunostaining of Myosin 7A (MYO7A), a hair cell marker, which again does not reveal any hair cell loss in *Anxa4^–/–^* mice (Figures 2B,C). Taken together, our present data suggest that the development and maintenance of cochlear hair cells are unaffected in *Anxa4^–/–^* mice.

The auditory function of *Anxa4^–/–^* mice was then evaluated by performing auditory brainstem response (ABR) measurements. No significant differences in ABR thresholds to either click or pure-tune stimuli were found between 2-month-old wild type and *Anxa4^–/–^* mice (Figures 2D,E). Similar results were obtained in *Anxa4^–/–^* mice up to age of 5 months (Figure 2F). To investigate whether *Anxa4^–/–^* mice show increased acoustic vulnerability, we exposed 2-month-old mice to 2–20 kHz noise at 110 dB SPL for 2 hours. ABR thresholds were measured before and after the noise exposure, which does not reveal any significant difference between wild type and *Anxa4^–/–^* mice (Figure 2G). Taken together, our data suggest that the auditory function is not affected in *Anxa4^–/–^* mice.

### VHCs and Vestibular Function Are Unaffected in *Anxa4* Knockout Mice

**FIGURE 3:**
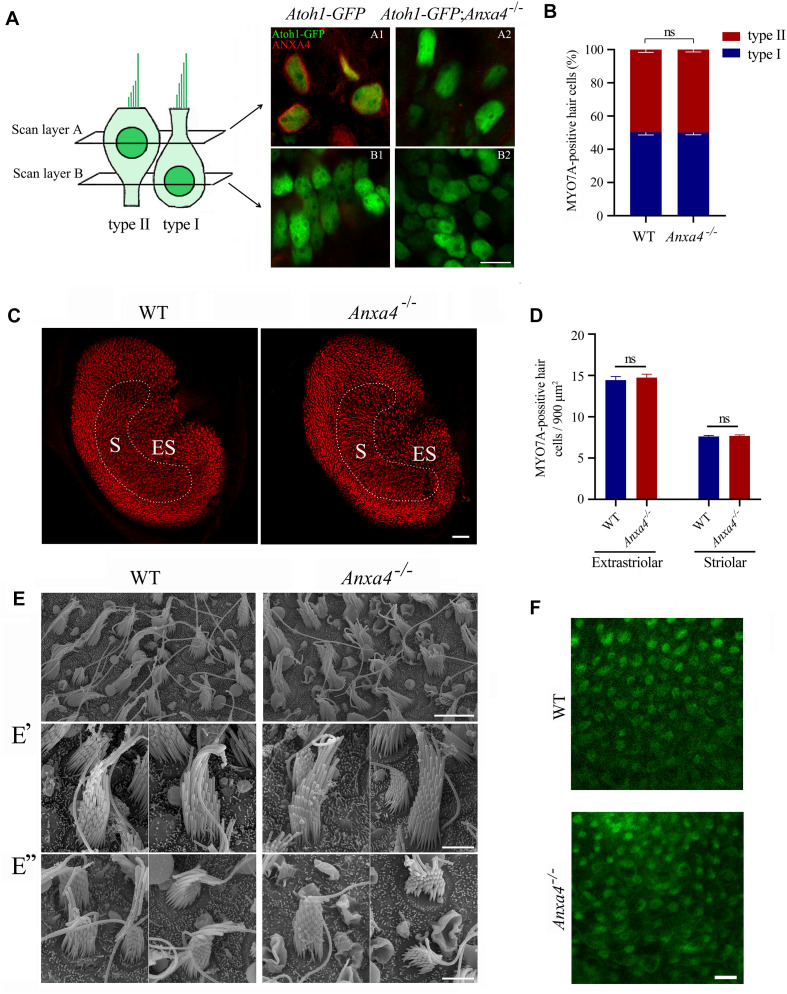
VHCs are unaffected in *Anxa4^–/–^* mice. **(A)** VHCs in the utricle of P60 *Atoh1-GFP* or *Atoh1-GFP*; *Anxa4^–/–^* mice were stained with anti-ANXA4 antibody (R&D Systems). Hair cells were labeled by nuclear GFP. Confocal microscopy images were taken at different levels as indicated. **(B)** VHCs in the utricle of P60 wild type or *Anxa4^–/–^* mice were stained with anti-MYO7A antibody, and numbers of type I and type II MYO7A-positive VHCs were calculated. **(C)** VHCs in the utricle of P60 wild type or *Anxa4^–/–^* mice were stained with TRITC-conjugated phalloidin and imaged with confocal microscope. **(D)** Numbers of striolar or extrastriolar phalloidin-positive VHCs were calculated according to the results from **(C)**. **(E)** Examination of utricle hair bundles of P60 wild type or *Anxa4^–/–^* mice using SEM. **(E’,E”)** Higher resolution SEM images of type I and type II VHCs, respectively. **(F)** FM1-43FX uptake by VHCs of utricle in P7 wild type or *Anxa4^–/–^* mice was examined using confocal microscope. The numbers of animals used in each groups in **(B,D)** are 3. Scale bars, 10 μm in **(A,F)**, 5 μm in **(E)**, 2 μm in **(E’,E”)**, 50 μm in **(C)**. ns, not significant.

Mammalian VHCs are divided into type I and type II according to their morphology, physiology and innervation patterns (Eatock et al., 1998; Deans, 2013). Morphologically, type I VHCs have flask-shaped cell bodies with nuclei above the supporting cell nuclei, whereas type II VHCs have cylindrical, goblet-like, or dumb-bell shaped cell bodies with nuclei close to the lumen (Pujol et al., 2014). Moreover, the stereocilia of type II VHCs are shorter than that of type I (Li et al., 2008). ANXA4 has been reported to be specifically expressed in type II VHCs (McInturff et al., 2018). By performing whole-mount immunostaining, we confirm that ANXA4 is expressed on the plasma membrane of type II VHCs in neonatal *Atoh1-GFP* mice but not *Atoh1-GFP*; *Anxa4^–/–^* mice (Figure 3A). However, the numbers of either VHC type are largely unaffected in *Atoh1-GFP*; *Anxa4^–/–^* mice (Figure 3A). Immunostaining using anti-MYO7A antibody and phalloidin staining further confirms that the percentage of different VHC types as well as the density of VHCs are not significantly different between wild type and *Anxa4^–/–^* mice at P60 (Figures 3B–D), suggesting that VHC development and maintenance is not affected by *Anxa4* disruption.

SEM was then employed to examine the morphology of VHC hair bundles of adult *Anxa4^–/–^* mice. The results show that in the utricle of P60 *Anxa4^–/–^* mice, hair bundles are morphologically normal compared with that of wild type mice (Figures 3E–E”). Furthermore, FM1-43FX uptake results show that MET function remains normal in *Anxa4^–/–^* VHCs (Figure 3F).

**FIGURE 4:**
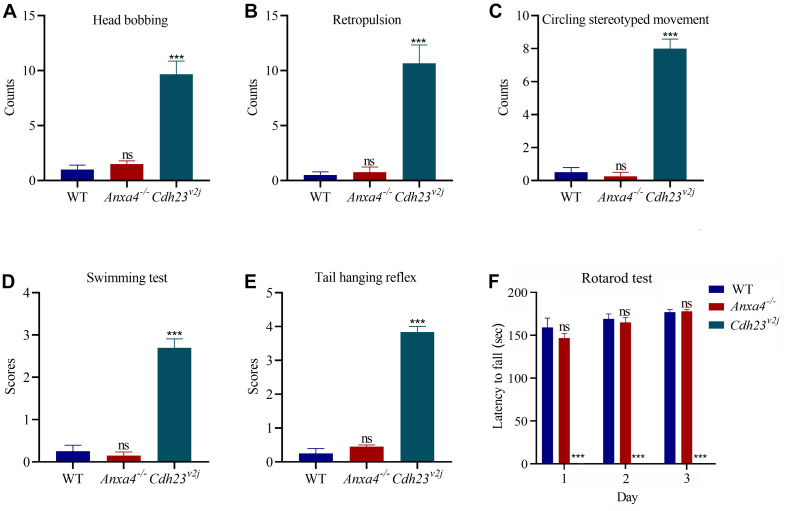
Vestibular function is unaffected in *Anxa4^–/–^* mice. The vestibular function of 2-month-old *Anxa4^–/–^* and wild type mice (*n* = 4) was evaluated by performing **(A)** head bobbing, **(B)** retropulsion, **(C)** circling stereotyped movement, **(D)** swimming, **(E)** tail hanging reflex, and **(F)** rotarod test. Five-month-old *Cdh23* mutant mice (*V*^2J^) (*n* = 3) were included as positive control. ns, not significant; ***P < 0.001.

The vestibular function of *Anxa4^–/–^* mice was evaluated by performing a series of examinations including head bobbing, retropulsion, circling stereotyped movement, swimming, tail hanging reflex, and rotarod test. *Cdh23* mutant mice (*V*^2J^) have been reported to show profound vestibular abnormalities (Di Palma et al., 2001), hence were included as a control to confirm the validity of the tests. The results do not reveal any significant difference between 2-month-old *Anxa4^–/–^* mice and wild type mice, suggesting that the vestibular function of *Anxa4^–/–^* mice is normal (Figures 4A–F).

### Other Annexin Family Members Are Expressed in Mouse Inner Ear

So far, our data show that ANXA4 is specifically expressed in inner ear hair cells, and is dispensable for hair cell development and function. ANXA4 belongs to the annexin protein family, which consists of 12 proteins (ANXA1-11, ANXA13) in vertebrates (Moss and Morgan, 2004). We wonder whether the lack of phenotype in *Anxa4^–/–^* mice is because of the expression of other annexins in the hair cells. RT-PCR was then performed to examine the expression of annexin family members, which shows that *Anxa1*, *Anxa2*, *Anxa5*, *Anxa6*, and *Anxa7* are all expressed in the inner ear of wild type mice (Supplementary Figure 3A). However, the expression of these annexin genes are not altered in *Anxa4^–/–^* mice, which is further confirmed by qPCR (Supplementary Figures 3A,B).

## Discussion

*Anxa4* has been shown to be specifically expressed in the cochlear and vestibular hair cells, suggesting that it might play an important role in the development and/or function of hair cells (Cai et al., 2015; McInturff et al., 2018). In the present work, we established *Anxa4* knockout mice to explore the role of ANXA4 in hair cells. Exon 3-6 of *Anxa4* gene is deleted in the knockout mice, which will disrupt the expression of ANXA4 protein. Furthermore, RT-PCR results reveal that this deletion results in degradation of *Anxa4* mRNA. Western blot and immunostaining using two different anti-ANXA4 antibodies confirm that ANXA4 protein is not present in the homozygous knockout mice.

Unexpectedly, our present data show that *Anxa4* gene disruption does not affect hair cell development and function. The morphology and MET function of both cochlear and vestibular hair cells are largely normal in *Anxa4* knockout mice. As a result, no abnormalities are detected by standard auditory and vestibular function tests in *Anxa4* knockout mice. One possibility is that ANXA4 is required for the function of hair cells under some specific conditions that are not examined in our present study. Alternatively, the function of ANXA4 might be compensated or substituted by other proteins such as other annexins.

Among all the annexin members, ANXA4 and Annexin A5 (ANXA5) are evolutionally closest to each other, and differ from most of the other annexins in their self-assembly ability on membranes with negatively charged phospholipids as well as the membrane curvature they induce (Concha et al., 1992; Kaetzel et al., 2001; Bouter et al., 2011; Crosby et al., 2013; Boye et al., 2018). ANXA5 has recently been shown to be highly expressed in the stereocilia as well as cell body of hair cells (Krey et al., 2016). Interestingly, similar to *Anxa4^–/–^* mice, *Anxa5^–/–^* mice are viable, fertile, and do not show any auditory and vestibular deficits (Brachvogel et al., 2003; Krey et al., 2016). No upregulation of *Anxa5* or *Anxa4* were detected in *Anxa4^–/–^* or *Anxa5^–/–^* mice, respectively, suggesting that there is no compensation between these two genes (Krey et al., 2016) (and the present study). However, this does not rule out the possibility of functional redundancy between ANXA4 and ANXA5 in the hair cells. Analysis of *Anxa4* and *Anxa5* double knockout mice will certainly help to address this question.

Interestingly, our present data show that ANXA4 is enriched at the tips of stereocilia besides localizing on the plasma membrane in cochlear hair cells. Similar stereocilia localization has also been reported for ANXA5 (Krey et al., 2016). The underlying mechanism of their transportation and function at the tips of stereocilia remain elusive. In the intestine, annexin A13 (ANXA13) is localized at the tips of microvilli, the actin-based cell protrusions similar to stereocilia (McCulloch et al., 2019). The function of ANXA13 at the tips of microvilli is still unknown, but this localization is consistent with a possible role in lumenal vesicle formation (McConnell et al., 2009). Identification of ANXA4/5-binding partners in the stereocilia as well as examination of the stereocilia phenotypes of *Anax4*/*Anxa5* double knockout mice will help us to learn more about their roles in the stereocilia.

## Data Availability Statement

The original contributions presented in the study are included in the article/Supplementary Material, further inquiries can be directed to the corresponding author/s.

## Ethics Statement

The animal study was reviewed and approved by the Animal Ethics Committee of Shandong University School of Life Sciences.

## Author Contributions

ZX: study concept and design. NL, YX, HD, and HZ: acquisition of data. NL, YX, HD, HZ, and ZX: analysis and interpretation of data. NL and ZX: drafting the manuscript. All the authors contributed to the article and approved the submitted version.

## Conflict of Interest

The authors declare that the research was conducted in the absence of any commercial or financial relationships that could be construed as a potential conflict of interest.
