# Supplementary material for: Annexin A4 Is Dispensable for Hair Cell Development and Function
Source: Front Cell Dev Biol. 2021 Jun 3;9:680155. doi: 10.3389/fcell.2021.680155 (PMC8209329; doi:10.3389/fcell.2021.680155)
Supplement: Supplementary file 1 [file Data_Sheet_1.pdf]

Annexin A4 is dispensable for hair cell development and function

Nana Li[1], Yuehui Xi[1], Haibo Du[1], Hao Zhou[1], Zhigang Xu[1,2*]

[1]Shandong Provincial Key Laboratory of Animal Cell and Developmental Biology, School of Life Sciences, Shandong University, Qingdao, Shandong 266237, China

[2]Shandong Provincial Collaborative Innovation Center of Cell Biology, Shandong Normal University, Jinan, Shandong 250014, China

*Author for correspondence (xuzg@sdu.edu.cn)

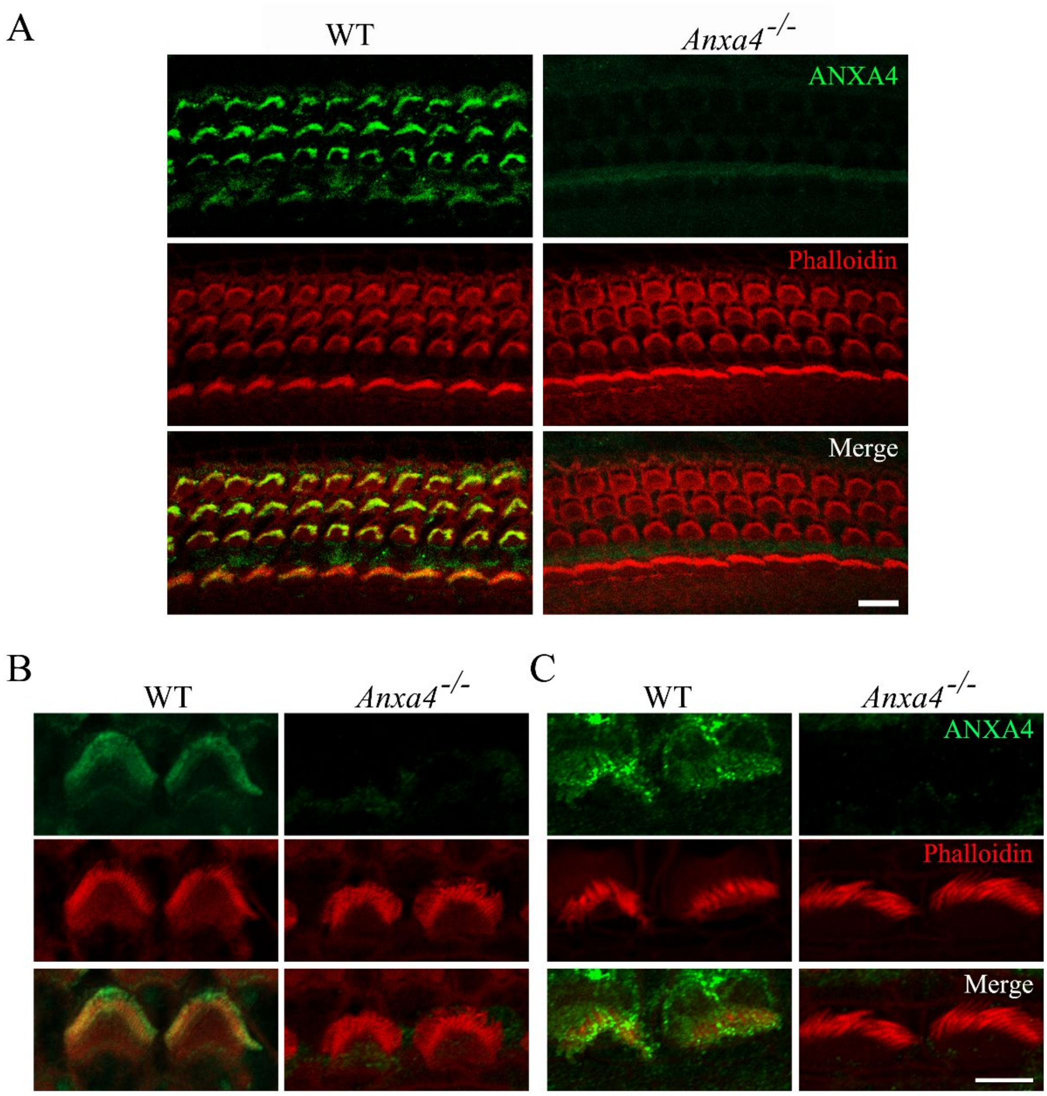

Supplementary Figure 1. ANXA4 immunoreactivity is localized in the stereocilia. Cochlear hair cell stereocilia of P7 wild type or *Anxa4*$^{-/-}$ mice were stained with anti-ANXA4 antibody (R&D Systems) and imaged using confocal microscope. Stereocilia F-actin core was visualized by staining with TRITC-conjugated phalloidin. Shown are images taken from the middle turn. (A) Lower resolution images show ANXA4 immunoreactivity in the stereocilia of OHCs and IHCs from wild type but not *Anxa4*$^{-/-}$ mice. (B) and (C) High resolution images show ANXA4 immunoreactivity at the stereociliary tips of OHCs (B) or IHCs (C) from wild type but not *Anxa4*$^{-/-}$ mice. Scale bars, 10 μm in (A), 5 μm in (B) and (C).

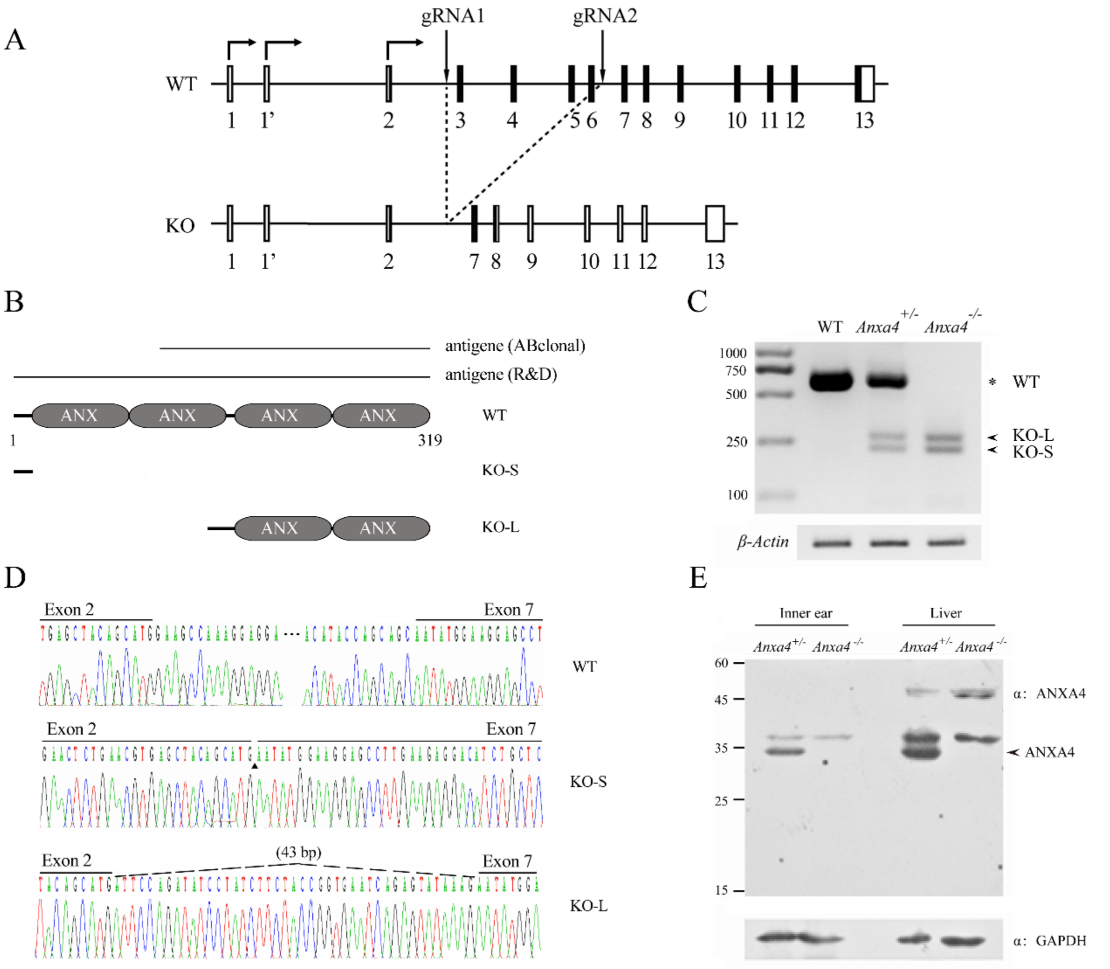

Supplementary Figure 2. Construction and validation of *Anxa4* knockout mice. (A) Schematic drawing of mouse *Anxa4* genomic structure and the strategy for construction of *Anxa4* knockout mice. Mouse *Anxa4* gene contains 13 exons and transcription could start from exon 1, exon 2, or an alternative exon 1 (exon 1'). Two gRNAs were used to delete exons 3-6. (B) Schematic drawing of the domain architecture of ANXA4 protein encoded by wild type (WT) or knockout transcripts (KO-S and KO-L). The regions recognized by the two anti-ANXA4 antibodies are indicated by solid lines. (C) Total RNA was extracted from the inner ear of P2 mice of different genotypes, and PCR was performed to examine the level of *Anxa4* mRNA. *β-actin* was used as an internal control. (D) The PCR products from (C) were purified and subjected to Sanger sequencing. (E)

Proteins were extracted from the inner ear or liver of P5 mice of different genotypes, and western blot was performed to examine the level of ANXA4 protein using an anti-ANXA4 antibody (ABclonal). GAPDH was used as an internal control.

A

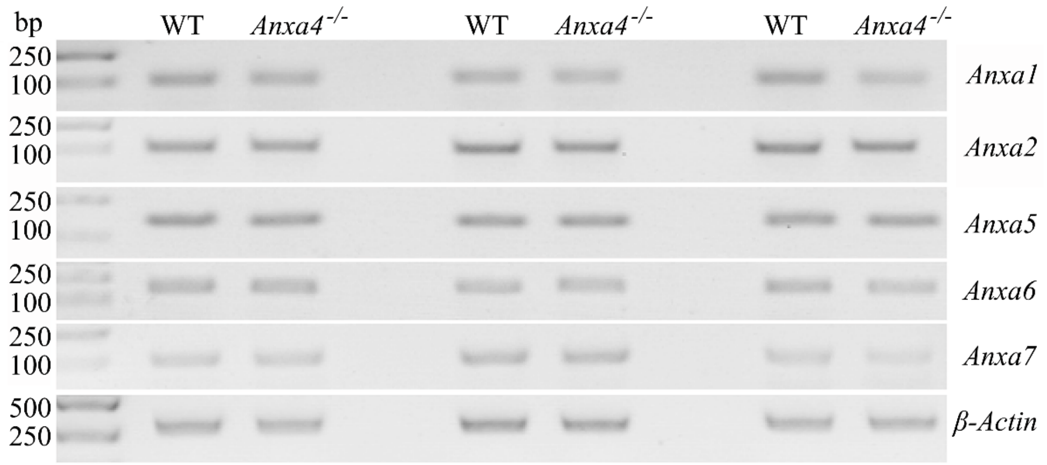

B

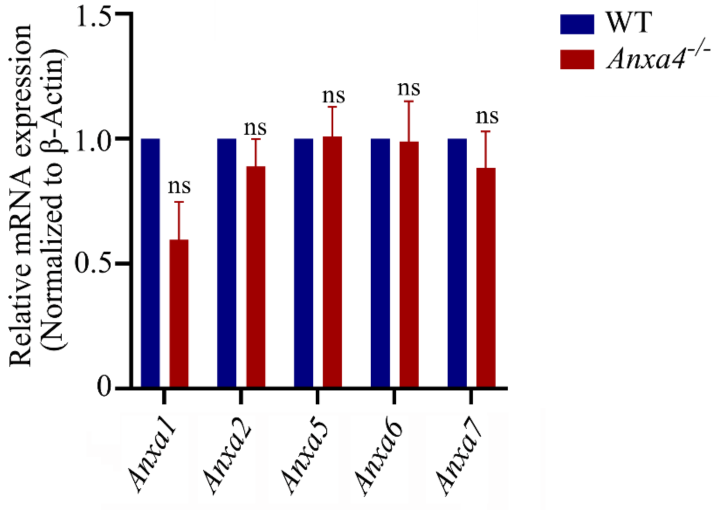

Supplementary Figure 3. Expression of other annexin genes in the inner ear of P2 wild type or *Anxa4*$^{-/-}$ mice was examined by performing RT-PCR (A) and qPCR (B). ns, not significant.
